# Trends and determinants of antimicrobial susceptibility of *Neisseria gonorrhoeae* in the Netherlands, 2007 to 2015

**DOI:** 10.2807/1560-7917.ES.2018.23.36.1700565

**Published:** 2018-09-06

**Authors:** Sanne HI Hofstraat, Hannelore M Götz, Alje P van Dam, Marianne AB van der Sande, Birgit HB van Benthem

**Affiliations:** 1 National Institute for Public Health and the Environment (RIVM), Centre for Infectious Disease Control, Bilthoven, the Netherlands; 2 Department of Infectious Disease Control, Municipal Public Health Service Rotterdam-Rijnmond, Rotterdam, the Netherlands; 3 Public Health Laboratory, Amsterdam Health Service, Amsterdam, the Netherlands; 4 Julius Centre for Health Sciences and Primary Care, University Medical Centre, Utrecht, the Netherlands; 5 Department of Public Health, Institute of Tropical Medicine, Antwerp, Belgium

**Keywords:** sexually transmitted infections, gonorrhoea, antimicrobial resistance

## Abstract

*Neisseria gonorrhoeae* antibiotic resistance surveillance is important to maintain adequate treatment. We analysed 2007–15 data from the Gonococcal Resistance to Antimicrobials Surveillance (GRAS), which currently includes 19 of 25 sexually transmitted infection (STI) centres in the Netherlands. **Methods**: From each patient with a gonorrhoea culture, the minimum inhibitory concentration (MIC) for several antibiotics was determined. Time trends were assessed by geometric means and linear regression of logarithmic MIC. Determinants for decreased susceptibility to ceftriaxone (MIC > 0.032 mg/L) and resistance to cefotaxime (MIC > 0.125 mg/L) and azithromycin (MIC > 0.5 mg/L) were assessed using stratified logistic regression. **Results**: 11,768 isolates were analysed. No ceftriaxone resistance was found. In 2015, 27 of 1,425 isolates (1.9%) were resistant to cefotaxime and 176 of 1,623 (10.9%) to azithromycin. Ceftriaxone susceptibility showed no trend (p = 0.96) during the study period, but cefotaxime MIC decreased (p < 0.0001) and azithromycin MIC increased (p < 0.0001) significantly. Concerning ceftriaxone, isolates of men who have sex with men (MSM) from 2013 (p = 0.0005) and 2014 (p = 0.0004) were significantly associated with decreased susceptibility. Significant determinants for cefotaxime resistance were having ≥ 6 partners for women (p = 0.0006). For azithromycin,****isolates from MSM collected in 2012 (p = 0.0035), 2013 (p = 0.012), and 2014 (p = 0.013), or from non-Dutch (p < 0.0001) or older (≥ 35 years; p = 0.01) MSM were significantly associated with susceptibility. Resistance in heterosexual men was significantly associated with being ≥ 25 years-old (p = 0.0049) or having 3–5 partners (p = 0.01). **Conclusions**: No ceftriaxone resistance was found, but azithromycin MIC increased in 2007–15. Resistance determinants could help with focused intervention strategies.

## Introduction

Gonorrhoea, caused by *Neisseria gonorrhoeae*, is one of the most common sexually transmitted infections (STI) and, with increasing resistance, a major public health concern globally [1]. Gonorrhoea is the second most common bacterial STI in the Netherlands. Under the national sentinel surveillance programme in STI centres, 5,391 cases were reported in 2015, and gonorrhoea was most prevalent among men who have sex with men (MSM): 10.7 per cent tested positive for gonorrhoea compared with 1.9 per cent and 1.6 per cent in heterosexual men and women respectively [2].

Third generation (3G) cephalosporins, such as ceftriaxone and cefixime (and cefotaxime in the Netherlands), are routinely used for the treatment of gonorrhoea in most countries. In the Netherlands, cefotaxime became the first-line therapy for gonorrhoea in 2003 and ceftriaxone in 2006 [3]. However, the susceptibility of gonococci to these cephalosporins has been decreasing and *N. gonorrhoeae* has developed antimicrobial resistance (AMR) to most drugs used for treatment. Several treatment failures for 3G cephalosporins have been reported; cefixime treatment failures have been verified in several countries [4-10]. In the Netherlands, treatment failure with cefotaxime has been reported once so far [11]. A 2012 European guideline subsequently advised to exclude cefixime and cefotaxime from the first-line antimicrobial treatment recommendations [12].

The present first-line treatment of choice in most countries worldwide is ceftriaxone, often combined in dual therapy with azithromycin [12-14]. In the Netherlands, it was decided in 2012 to only advise single therapy ceftriaxone (500 mg) [15], which is in accordance with the 2016 World Health Organization (WHO) guidelines [14]. However, outside of the Netherlands, resistance and treatment failures have been described for both drugs [16-22]. The first high-level azithromycin-resistant gonorrhoea was reported in the United Kingdom (UK) in 2015 [23]. Moreover, the first treatment failure on dual therapy of azithromycin and ceftriaxone was reported in 2016 in the UK [24]. This rapid development of *N. gonorrhoeae* resistance to antibiotics threatens effective treatment. Targeted surveillance of new resistance patterns and insights, both into their spread into sexual networks and in their determinants are essential to control this trend.

To maintain adequate and updated treatment and prevention guidelines, the Gonococcal Resistance to Antimicrobials Surveillance (GRAS) was implemented within the Dutch STI sentinel surveillance network in July 2006. The STI sentinel surveillance system has national coverage and all consultations and corresponding diagnoses are reported online to the National Institute of Public Health and the Environment of the Netherlands (RIVM) for surveillance purposes, a process that is facilitated by a web-based application (SOAP) [2]. In this study, results of the GRAS surveillance are analysed, combining epidemiological and microbiological data to focus on trends in antimicrobial susceptibility of *N. gonorrhoeae* in the Netherlands between 2007 and 2015. We also identified determinants associated with decreased ceftriaxone susceptibility as well as azithromycin and cefotaxime resistance.

## Methods

GRAS includes data from STI centres across the Netherlands and from laboratories connected to STI centres, as well as laboratories that also test other patients. The STI centres provide free STI testing and care to people in specified high-risk groups, e.g. MSM and people < 25 years-old [2]. Patients who are not part of a specified high-risk group are referred to their general practitioner (GP) for STI testing and care, which is not free for low-risk groups. Currently, GRAS includes 19 of a total 25 STI centres in the Netherlands.

### Antimicrobial susceptibility testing

From each client who tests positive for gonorrhoea, a sample is requested for culture and susceptibility testing. For GRAS, minimum inhibitory concentration (MIC) values are collected and reported in SOAP for each diagnosed gonorrhoea patient with a positive culture. The antimicrobial susceptibility of gonococcal isolates is tested locally at laboratories related to the STI centres. The isolates are tested for azithromycin, cefotaxime, ceftriaxone, ciprofloxacin and spectinomycin using Etest (bioMérieux, Marcy l'Etoile, France) determining the MIC. Azithromycin and ceftriaxone were included in GRAS in 2011. Because GRAS started in July 2006 there are many missing data that year, therefore the analyses in this study were performed on results from 2007 up to and including 2015.

### Study population

The study population consisted of all patients of participating STI centres who were diagnosed with a *N. gonorrhoeae* infection and where antimicrobial susceptibility data were available. When patients were positive for gonorrhoea on multiple sites and more than one culture was obtained, only one culture was included in GRAS. Laboratories were requested to report the isolate with the highest MIC and in case of equal MICs, the European Centre for Disease prevention and Control (ECDC) guideline for European Gonococcal Antimicrobial Surveillance Programme (EURO-GASP) reporting was used [25] giving the following order of preference when multiple sites were infected. For males: pharyngeal, rectal, urethral, other; for females: pharyngeal, cervical, other anogenital (high vaginal swab/rectal/urethral), other.

For each visitor an anonymous report is submitted containing epidemiological and clinical data, as well as test results on a wide range of STI. Ethnicity is based on (parental) country of birth. A person is defined as native Dutch if both parents were born in the Netherlands. A test of cure is not generally recommended.

Since June 2014, an individual ID number is available in SOAP, which enables identification of individual patients and their subsequent consultations. All analyses were performed using isolates collected during individual consultations. Patients having multiple sequential infections could be included for each infectious episode.

As this study uses national data from the GRAS surveillance, the data mentioned in Wind et al. [26] and Heymans et al. [27], which is specific for Amsterdam, is reused in a wider context in this report as part of the dataset.

### Data analysis

Descriptive analyses of the study population were performed. The chi-squared test was used to assess the significance in differences among groups (MSM, heterosexual men and women). Time trends were assessed by calculating mean MICs as geometric means per year and by performing a linear regression analysis of the logarithmic MICs. The criteria used to define resistance are those used by the European Committee on Antimicrobial Susceptibility Testing (EUCAST) [28]. Because there were very few strains that reached the 0.125 mg/L threshold of ceftriaxone resistance, for risk factor analysis, strains with a MIC > 0.032 mg/L for ceftriaxone were regarded as having a decreased susceptibility (the epidemiological cut-off value according to EUCAST [28]). Determinants for decreased susceptibility for ceftriaxone (MIC > 0.032 mg/L) and resistance for cefotaxime (MIC > 0.125 mg/L) and azithromycin (MIC > 0.5 mg/L) were identified using logistic regression analyses. Since sexual orientation is highly correlated with many other variables, we performed separate analyses for MSM, heterosexual men and women. Multivariable analyses using backward selection were performed using all variables with clinical and statistical (p < 0.2) importance in the univariable analyses. In the multivariable analysis, statistical significance was determined as p < 0.05. As we wanted to study trends over time, year of infection was always included in the model. All statistical analyses were performed using SAS software, version 9.4 (VMware, Inc).

## Results

Between 2007 and 2015 susceptibility testing for *N. gonorrhoeae* was performed for 11,768 isolates, covering 41.8% of all patients (n = 28,175) diagnosed with gonorrhoea in participating STI centres in that period. Before June 2014, no information about sequential infections in individual patients was available. Since June 2014, the majority of patients (2,162/2,550; 84.8%) were included only once; 163 of 2,550 patients (6.4%) were included twice; 103 of 2,550 patients (4.0%) were included three times and 122 of 2,550 patients (4.8%) were included with four to eight consultations. Of the 388 patients who were included more than once 357 (92.0%) were MSM.

### Baseline characteristics of patients

Eighty-five per cent of the isolates included were collected from men (10,014/11,768). Sixty-four per cent (7,488/11,768) of the isolates were from MSM and 42.4% (4,992/11,768) were people of Dutch origin (Table 1). Table 1 also shows that almost half of the MSM with an isolate were older than 35 years (3,640/7,488) whereas most women (66.1%) were younger than 25 years of age (1,159/1,754). Fourteen per cent of women (252/1,754) worked as commercial sex workers (CSW) and 12.2% of heterosexual men (309/2,526) were a client of a CSW. Most isolates came from the urban regions of the Netherlands; the region of Amsterdam accounted for 61.3% (7,214/11,768) of all isolates between 2007 and 2015 (Amsterdam accounted for 42.0% of all gonorrhoea diagnoses). Patients with a positive culture differed from patients with a negative culture or without culture regarding demographic characteristics. Supplement 1 shows the characteristics of all gonorrhoea patients from Dutch STI centres with and without an isolate for susceptibility testing. Patients with a positive culture were more often male, older, MSM, non-Dutch and human immunodeficiency virus (HIV) positive (data not shown). The total percentage of isolates available for susceptibility testing has decreased over time, from 61.0% of all diagnoses in 2007 to 26.7% in 2015, due to an increase in the total number of tests, the limitations of the GRAS surveillance where only one isolate per patient can be reported and negative cultures. Overall, about half of the anal and cervical cultures was negative (50.3% (3,288/6,539) and 51.1% (1,214/2,376) respectively), the percentage of negative urethral cultures was slightly lower (39.8%; 2,894/7,267) while the highest percentage of negative cultures was for oral cultures (72.2%, 3,186/4,410). 

**Table 1:** Characteristics of gonorrhoea patients from Dutch sexually transmitted infection centres with culture-positive *Neisseria gonorrhoeae*, by sexual orientation, 2007–2015 (n = 11,768 consultations)

Characteristic	MSM (n = 7,488)		Heterosexual men (n = 2,526)		Women (n = 1,754)		Total (N = 11,768)	
	n	%	n	%	n	%	n	%
**Year of diagnosis**								
2007	572	7.6	227	9.0	133	7.6	932	7.9
2008	635	8.5	184	7.3	110	6.3	929	7.9
2009	694	9.3	247	9.8	156	8.9	1,097	9.3
2010	762	10.2	301	11.9	171	9.8	1,234	10.5
2011	837	11.2	351	13.9	239	13.6	1,427	12.1
2012	980	13.1	394	15.6	238	13.6	1,612	13.7
2013	1,006	13.4	294	11.6	247	14.1	1,547	13.2
2014	1,005	13.4	299	11.8	244	13.9	1,548	13.2
2015	997	13.3	229	9.1	216	12.3	1,442	12.3
**Age (years)**								
< 25	1247	16.7	1017	40.3	1159	66.1	3,423	29.1
25–35	2601	34.7	939	37.1	375	21.4	3,915	33.3
≥ 35	3,640	48.6	570	22.6	220	12.6	4,430	37.6
**Ethnicity**								
Dutch	3,189	42.6	1,030	40.8	773	44.1	4,992	42.4
Non-Dutch	4,299	57.4	1,496	59.2	981	55.9	6,776	57.6
**CSW (for MSM or women)**								
No	7,281	97.2	NA	NA	1,498	85.4	8,779	74.6	
Yes, in past 6 months	176	2.4			252	14.4	428	3.6	
Unknown	31	0.4			4	0.2	35	0.3	
**Client of CSW (for men including MSM)**								
No	7,362	98.3	2,210	87.5	NA	NA	9,572	81.3
Yes, in past 6 months	74	1.0	309	12.2			383	3.3
Unknown	52	0.7	7	0.3			59	0.5
**New HIV infection**								
No	7,250	96.8	2,516	99.6	1,752	99.9	11,518	97.9
Yes	238	3.2	10	0.4	2	0.1	250	2.1

CSW: commercial sex worker; MSM: men who have sex with men.

### Antimicrobial susceptibility over time

**Figure 1:**
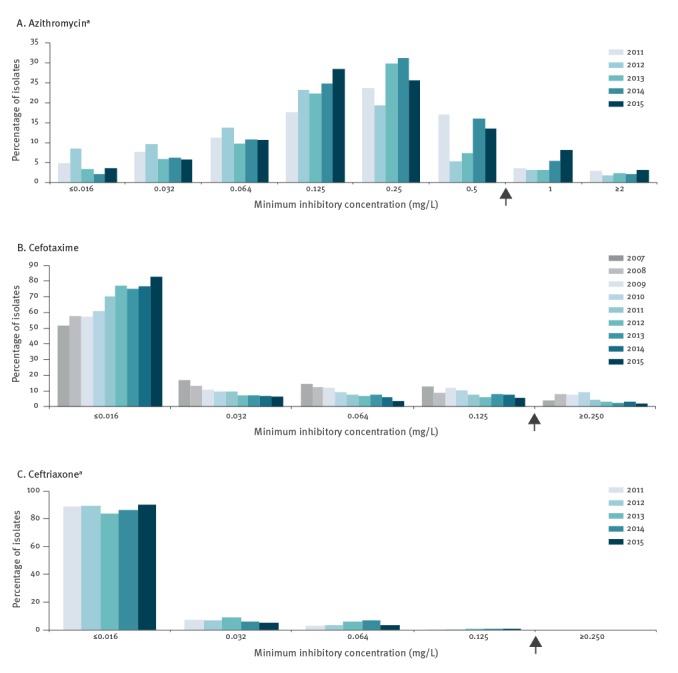
Distribution of minimum inhibitory concentration values for azithromycin^a^, cefotaxime and ceftriaxone^a^ susceptibility of *Neisseria gonorrhoeae* from patients of Dutch sexually transmitted infection centres, 2007–2015

EUCAST: European Committee on Antimicrobial Susceptibility Testing; GRAS: Gonococcal Resistance to Antimicrobials Surveillance.

Figure 1 shows the distribution of MIC values over time for azithromycin (Figure 1A), cefotaxime (Figure 1B), and ceftriaxone (Figure 1C). For azithromycin, the highest proportion of isolates had a MIC of 0.250 mg/L. The proportion of isolates with a MIC of 1 mg/L appears to have increased over the last few years, but the proportion of isolates with a MIC ≥ 2 mg/L remained stable with 3.3% (42/1,265) of isolates in 2011 and 3.0% (49/1,623) of isolates in 2015 . For cefotaxime, the biggest proportion of isolates tested had a MIC ≤ 0.016 mg/L. The percentage of strains with higher MICs appears to have decreased over time, as well as the proportion of isolates with resistance that decreased from 4.0% (37/927) in 2007 to 1.9% (27/1,425) in 2015. When tested for ceftriaxone, most isolates also showed a MIC ≤ 0.016 mg/L. In 2015, four of 1,446 isolates (0.3 %) showed a MIC of 0.125 mg/L for ceftriaxone compared to two of 1,548 (1.3 %) isolates in 2014. In contrast to cefotaxime, the percentage of strains with an increased MIC for ceftriaxone (≥ 0.032 mg/L) seems to have increased from 3.5% (44/1,262) in 2011 to 7.5% (115/1,528) in 2014, although in 2015 it decreased again to 4.5% (64/1,416). 

The arrows indicate the threshold for antimicrobial resistance according to EUCAST guidelines.

^a ^Azithromycin and ceftriaxone were included in GRAS in 2011.

Over time, the mean MIC for cefotaxime decreased significantly (p < 0.0001) from 0.020 mg/L in 2007 to 0.008 mg/L in 2015 (Table 2). For ceftriaxone, the mean MIC also decreased slightly, from 0.006 mg/L in 2011 to 0.004 mg/L in 2015, but no trend was found (p = 0.96). There was a significant increase (p < 0.0001) in mean MIC for azithromycin, from 0.160 mg/L in 2011 to 0.173 mg/L in 2015.

**Table 2:** Geometric mean minimum inhibitory concentration per year for cefotaxime, ceftriaxone and azithromycin, found at Dutch sexually transmitted infection centres, 2007–2015

Year	Geometric mean minimum inhibitory concentration (mg/L) (95%CI)			Total number of isolates
	Cefotaxime	Ceftriaxone^a^	Azithromycin^a^	
2007	0.0195 (0.0180–0.0211)	NA^a^	NA^a^	932
2008	0.0171 (0.0157–0.0187)	NA^a^	NA^a^	929
2009	0.0163 (0.0149–0.0178)	NA^a^	NA^a^	1,097
2010	0.0143 (0.0131–0.0156)	NA^a^	NA^a^	1,234
2011	0.0099 (0.0092–0.0107)	0.0063 (0.0059–0.0066)	0.1595 (0.1492–0.1705)	1,427
2012	0.0089 (0.0084–0.0095)	0.0043 (0.0041–0.0046)	0.1058 (0.0994–0.1125)	1,612
2013	0.0096 (0.0090–0.0103)	0.0058 (0.0055–0.0062)	0.1518 (0.1430–0.1612)	1,547
2014	0.0097 (0.0091–0.0103)	0.0056 (0.0053–0.0059)	0.1726 (0.1641–0.1816)	1,548
2015	0.0083 (0.0078–0.0088)	0.0043 (0.0041–0.0046)	0.1729 (0.1630–0.1833)	1,442

CI: confidence interval; GRAS: Gonococcal Resistance to Antimicrobials Surveillance; NA: not available.

^a ^Azithromycin and ceftriaxone were included in GRAS in 2011.

### Antimicrobial resistance according to European committee on antimicrobial susceptibility testing

**Figure 2:**
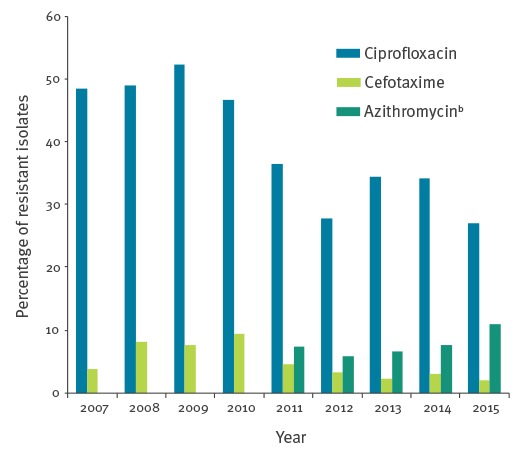
Yearly percentages of *Neisseria gonorrhoeae* isolates resistant^a^ to ciprofloxacin, cefotaxime and azithromycin at Dutch sexually transmitted infection centres, 2007–2015

EUCAST: European Committee on Antimicrobial Susceptibility Testing; GRAS: Gonococcal Resistance to Antimicrobials Surveillance.

^a ^Resistant according to EUCAST breakpoints.

^b ^Azithromycin was included in GRAS in 2011.

Figure 2 shows the percentage of isolates that were resistant to azithromycin, cefotaxime and ciprofloxacin according to EUCAST breakpoints [28]. No resistance to ceftriaxone was observed. The highest proportion of isolates with resistance was reported for ciprofloxacin; however, this seems to have decreased from 52% of isolates (574/1,095) in 2009 to 27% (391/1,426) in 2015. The proportion of isolates with resistance to cefotaxime appears to have decreased from 10.1% (114/1,129) in 2010 to 1.9% (27/1,425) in 2015 and the proportion of isolates resistant to azithromycin has increased from 5.8% (80/1,369) in 2012 to 10.9% (176/1,623) in 2015.

### Multiple drug resistance

Twenty-three isolates (6.3%; 23/366) that showed a decreased susceptibility for ceftriaxone (MIC > 0.032 mg/L) also were resistant for azithromycin according to EUCAST breakpoints and 118 of 366 isolates (32.2%) that had a decreased susceptibility for ceftriaxone were also resistant for cefotaxime (Table 3). For cefotaxime-resistant isolates, resistance for azithromycin was found in 18 of 532 isolates (3.4%). In total, 10 isolates were resistant for azithromycin and cefotaxime and less susceptible to ceftriaxone between 2007 and 2015. Highest frequencies of strains with decreased susceptibility and resistance to all antibiotics were seen in MSM (Table 3).

**Table 3:** Number of isolates with decreased ceftriaxone susceptibility or resistance to cefotaxime and azithromycin by sexual orientation, Netherlands, 2007–2015

Antibiotics	MSM (n = 7,488)		Heterosexual men (n = 2,526)		Women (n = 1,754)	
	N	%	N	%	N	%
Ceftriaxone^a^	0	0.0	0	0.0	0	0.0
Ceftriaxone (MIC > 0.032 mg/L)^b^	294	3.9	30	1.2	42	2.4
Cefotaxime^a^	410	5.5	70	2.8	52	3.0
Azithromycin^a^	428	5.7	64	2.5	50	2.9
Azithromycin (MIC > 1 mg/L)^b^	152	2.0	19	0.8	16	0.9
Cefotaxime^a^ + Azithromycin^a^	11	0.2	2	0.1	5	0.3
Ceftriaxone (MIC > 0.032 mg/L)^b^ + Cefotaxime^a^	86	1.1	15	0.6	17	1.0
Ceftriaxone (MIC > 0.032 mg/L)^b^ + Azithromycin^a^	17	0.2	2	0.1	4	0.2
Azithromycin^a^ + Cefotaxime^a^ + Ceftriaxone (MIC > 0.032 mg/L)^b^	6	0.08	1	0.0	3	0.2

EUCAST: European Committee on Antimicrobial Susceptibility Testing; MIC: minimum inhibitory concentration; MSM: men who have sex with men.

^a^ Resistant according to EUCAST breakpoints: ceftriaxone resistant (MIC > 0.125 mg/L); cefotaxime resistant (MIC > 0.125 mg/L); azithromycin resistant (MIC > 0.5 mg/L).

^b^ Decreased susceptibility according to breakpoint indicated in parentheses.

### Determinants of cefotaxime resistance (MIC > 0.125mg/L)

#### Men who have sex with men

Among MSM, being a client of a CSW in last 6 months was found to be significantly associated (p = 0.007) with cefotaxime resistance (adjusted odds ratio (aOR): 3.1) (Table 4).

**Table 4:** Univariable and multivariable analyses of cefotaxime resistance^a^ of *Neisseria gonorrhoeae* in patients of Dutch sexually transmitted infection centres, by sex/sexual orientation, 2007–2015 (n = 11,643 isolates)

Characteristics	Univariable analyses OR (95%CI)			Multivariable analyses aOR (95%CI)		
	MSM	Heterosexual men	Women	MSM	Heterosexual men	Women
**Year of diagnosis**						
2008 (vs 2007)	2.6 (1.6–4.2)	0.5 (0.2–1.8)	1.2 (0.2–6.1)	1.4 (0.4–5.4)	< 0.001 (< 0.001–> 999)	1.0 (0.2–5.4)
2009 (vs 2007)	2.3 (1.4–3.6)	1.0 (0.4–2.6)	2.0 (0.5–7.9)	2.0 (0.5–7.6)	0.6 (0.1–3.5)	1.6 (0.4–6.4)
2010 (vs 2007)	3.3 (2.1–5.2)	0.8 (0.3–2.1)	1.3 (0.3–5.5)	4.2 (1.2–14.5)	0.6 (0.1–3.4)	1.1 (0.2–6.0)
2011 (vs 2007)	1.2 (0.8–2.1)	0.9 (0.4–2.2)	1.1 (0.3–4.5)	1.1 (0.3–3.8)	0.8 (0.2–3.5)	0.9 (0.2–4.6)
2012 (vs 2007)	0.9 (0.5–1.5)	0.5 (0.2–1.3)	0.8 (0.2–3.4)	0.8 (0.3–2.8)	0.4 (0.1–1.9)	0.8 (0.1–4.3)
2013 (vs 2007)	0.5 (0.3–0.9)	0.6 (0.2–1.7)	0.9 (0.2–3.9)	0.5 (0.1–1.6)	0.5 (0.1–2.3)	0.8 (0.2–4.2)
2014 (vs 2007)	0.6 (0.4–1.1)	0.7 (0.3–1.8)	2.2 (0.6–8.0)	0.6 (0.2–1.9)	0.6 (0.1–2.7)	2.1 (0.5–9.3)
2015 (vs 2007)	0.4 (0.2–0.8)	0.1 (0.0–0.9)	1.5 (0.4–5.9)	0.4 (0.1–1.4)	0.1 (0.0–0.9)	1.3 (0.3–6.5)
**Age (years)**						
25–35 (vs < 25)	1.3 (0.9–1.8)	1.8 (1.0–3.4)	2.3 (1.2–4.4)	NA^b^	NA^b^	NA^b^
≥ 35 (vs < 25)	1.4 (1.0–1.9)	3.3 (1.8–6.1)	3.5 (1.8–7.0)			
**Other**						
Non-Dutch ethnicity^c^	0.6 (0.5–0.7)	0.8 (0.5–1.2)	1.3 (0.7–2.2)	NA^b^	NA^b^	NA^b^
CSW in last 6 months (MSM or women)	1.2 (0.6–2.2)	NA	3.3 (1.8–5.9)	NA^b^	NA^b^	NA^b^
Client of CSW in last 6 months	1.8 (0.8–4.0)	1.7 (0.9–3.1)	NA	3.1 (1.4–7.2)^d^	NA^b^	NA^b^
Previous GO/chlamydia/syphilis	1.3 (1.0–1.6)	0.9 (0.5–1.8)	0.8 (0.4–1.6)	NA^b^	NA^b^	NA^b^
3–5 partners in last 6 months (vs 0–2)	1.1 (0.8–1.6)	0.9 (0.5–1.8)	0.9 (0.4–2.4)	NA^b^	NA^b^	0.9 (0.3–2.3)
≥ 6 partners in last 6 months (vs 0–2)	1.1 (0.8–1.5)	1.5 (0.8–2.9)	4.4 (2.3–8.7)			3.5 (1.7–7.0)
Casual partner	1.0 (0.7–1.4)	2.3 (1.1–4.9)	1.1 (0.5–2.2)	NA^b^	NA^b^	NA^b^
Notified of an STI by partner	0.9 (0.7–1.1)	0.3 (0.1–0.9)	1.3 (0.7–2.4)	NA^b^	0.1 (0.0–0.9)^d^	NA^b^
No condom use at last contact	0.8 (0.6–1.1)	0.5 (0.3–0.9)	0.8 (0.4–1.5)	NA^b^	0.4 (0.2–0.8)^d^	NA^b^
Urethral/cervical *Neisseria gonorrhoeae* infection	1.0 (0.8–1.3)	NA^e^	0.5 (0.3–0.8)	NA^b^	NA^b^	NA^b^
Anal *Neisseria gonorrhoeae* infection	0.8 (0.7–1.0)	NA^f^	1.4 (0.8–2.4)	NA^b^	NA^b^	NA^b^
Oral *Neisseria gonorrhoeae* infection	1.0 (0.8–1.2)	NA^f^	2.8 (1.4–4.8)	NA^b^	NA^b^	2.1 (1.2–3.8)
Concurrent Ct infection	1.1 (0.8–1.3)	0.4 (0.2–0.7)	0.4 (0.2–0.8)	NA^b^	0.4 (0.2–0.9)	NA^b^

aOR: adjusted odds ratio; CI: confidence interval; CSW: commercial sex worker; EUCAST: European Committee on Antimicrobial Susceptibility Testing; GO: gonorrhoea; MIC: minimum inhibitory concentration; MSM: men who have sex with men; NA: not applicable; OR: odds ratio; STI: sexually transmitted infection.

^a ^Resistant according to EUCAST breakpoint (MIC > 0.125 mg/L).

^b^ Multivariable analyses using backward selection were only performed for variables with clinical and statistical importance (p < 0.2) in the univariable analyses.

^c ^A person with both parents born in the Netherlands was considered to be of Dutch ethnicity.

^d ^Factor not included in final model that excluded Amsterdam data.

^e ^Number of cases in the different categories not large enough to perform analysis.

^f^ No samples available for these sites in heterosexual men.

#### Heterosexual men

Not using a condom during the last sexual contact (aOR: 0.4; p = 0.0049), receiving notification for an STI by a partner (aOR: 0.1; p = 0.035), 2015 as year of diagnosis (aOR: 0.1; p = 0.04, compared with 2007 and having a *Chlamydia trachomatis* co-infection (aOR: 0.4; p = 0.019) were significantly associated with cefotaxime susceptibility.

#### Women

Multivariable analysis showed a significant association between cefotaxime resistance and oral infection (aOR: 2.1; p = 0.01), and having six or more partners (aOR: 3.5; p = 0.0006, compared with 0–2 partners).

### Determinants of decreased ceftriaxone susceptibility (MIC > 0.032 mg/L)

#### Men who have sex with men

Table 5 shows that in the multivariable logistic regression model decreased ceftriaxone susceptibility was significantly associated with 2013 and 2014 as year of diagnosis (aOR: 2.1; p = 0.0005; aOR: 2.1; p = 0.0004, compared with 2011), and non-Dutch ethnicity (aOR: 1.9; p = 0.0004). Urethral and anal infections were found to be significantly associated with ceftriaxone susceptibility (aOR: 0.5; p < 0.0001; aOR: 0.6; p < 0.0001).

**Table 5:** Univariable and multivariable analyses of decreased ceftriaxone susceptibility^a^ of *Neisseria gonorrhoeae* in patients of Dutch sexually transmitted infection centres, by sex/sexual orientation, 2011–2015 (n = 6,884 isolates)

Characteristics	Univariable analyses OR (95%CI)			Multivariable analyses aOR (95%CI)
	MSM	Heterosexual men	Women	MSM
**Year of diagnosis**				
2012 (vs 2011)	1.1 (0.7–1.8)	0.9 (0.3–2.8)	0.6 (0.1–3.2)	1.1 (0.7–1.7)
2013 (vs 2011)	2.1 (1.4–3.2)	1.2 (0.4–3.9)	1.4 (0.4–5.2)	2.1 (1.4–3.2)
2014 (vs 2011)	2.0 (1.3–3.0)	1.5 (0.5–4.3)	4.8 (1.6–14.4)	2.1 (1.4–3.2)
2015 (vs 2011)	1.1 (0.7–1.7)	0.6 (0.2–2.6)	3.4 (1.1–10.7)	1.2 (0.7–1.8)
**Age (years)**				
25–35 years (vs < 25 years)	0.8 (0.6–1.1)	2.2 (0.8–5.9)	1.1 (0.5–2.5)	NA^b^
≥ 35 years (vs < 25 years)	0.9 (0.6–1.2)	5.4 (2.0–14.4)	4.0 (1.9–8.2)	NA^b^
**Other**				
Non-Dutch ethnicity^c^	2.0 (1.4–2.9)	0.8 (0.3–1.7)	2.3 (1.1–5.1)	1.9 (1.3–2.7)^d^
CSW in last 6 months (MSM or women)	0.7 (0.3–1.7)	NA	4.2 (2.2–8.1)	NA^b^
Client of CSW in last 6 months	1.7 (0.6–4.2)	1.3 (0.4–3.9)	NA	NA^b^
Previous GO/chlamydia/syphilis	1.1 (0.9–1.5)	0.4 (0.1–1.6)	1.0 (0.8–2.1)	NA^b^
3–5 partners in last 6 months (vs 0–2)	0.8 (0.5–1.2)	1.2 (0.4–3.0)	1.7 (0.7–4.2)	NA^b^
≥ 6 partners in last 6 months (vs 0–2)	0.9 (0.6–1.2)	0.4 (0.2–1.6)	6.2 (3.0–12.8)	
Casual partner	1.2 (1.0–1.6)	2.7 (1.1–6.7)	1.1 (0.6–2.1)	NA^b^
Notified of an STI by partner	1.1 (0.8–1.4)	0.6 (0.2–1.6)	1.2 (0.6–2.2)	NA^b^
No condom use at last contact	0.7 (0.6–0.9)	0.4 (0.2–0.8)	0.4 (0.2–0.8)	NA^b^
Urethral/cervical *Neisseria gonorrhoeae* infection	0.6 (0.5–0.8)	NA^e^	0.4 (0.2–0.7)	0.5 (0.4–0.7)^d^
Anal *Neisseria gonorrhoeae* infection	0.8 (0.6–1.0)	NA^f^	2.2 (1.2–4.2)	0.6 (0.4–0.8)^d^
Oral *Neisseria gonorrhoeae* infection	1.2 (0.9–1.5)	NA^f^	2.0 (1.1–3.8)	NA^b^
Concurrent Ct infection	0.9 (0.7–1.2)	0.4 (0.1–0.9)	0.6 (0.3–1.2)	NA^b^

aOR: adjusted odds ratio; CI: confidence interval; CSW: commercial sex worker; EUCAST: European Committee on Antimicrobial Susceptibility Testing; GO: gonorrhoea; MIC: minimum inhibitory concentration; MSM: men who have sex with men; NA: not applicable; OR: odds ratio; STI: sexually transmitted infection.

For heterosexual men and women, only univariable analyses were performed because of the small number of isolates with an MIC > 0.032 mg/L.

^a ^Decreased ceftriaxone susceptibility according to breakpoint (MIC > 0.032 mg/L).

^b^ Multivariable analyses using backward selection were only performed for variables with clinical and statistical importance (p < 0.2) in the univariable analyses.

^c ^A person with both parents born in the Netherlands was considered to be of Dutch ethnicity.

^d ^Factor not included in final model that excluded Amsterdam data.

^e ^Number of cases in the different categories not large enough to perform analysis.

^f^ No samples available for these sites in heterosexual men.

For heterosexual men and women, only univariable analyses were performed because of the small number of isolates with an MIC > 0.032 mg/L. 

#### Heterosexual men

Univariable analyses show that being over 35 years of age (OR: 5.4; p = 0.0007) and having a casual sex partner (OR: 2.7; p = 0.0291) were associated with a decreased susceptibility to ceftriaxone among heterosexual men. Having a co-infection with *C. trachomatis* (OR: 0.4; p = 0.0251) and not using a condom at one’s last sexual encounter (OR: 0.4; p = 0.0119) were associated with susceptibility to ceftriaxone.

#### Women

In the univariable analysis, decreased susceptibility to ceftriaxone was associated with 2014 and 2015 as year of diagnosis (OR: 4.8; p = 0.0048; OR: 3.4; p = 0.0368, compared with 2011), being over 35 years-old (OR: 4.0; p = 0.0002), a non-Dutch ethnicity (OR: 2.3; p = 0.0338), working as a CSW (OR: 4.2; p < 0.0001), having six or more partners (OR: 6.2; p < 0.0001, compared with 0–2 partners), and anal and oral infections (OR: 2.2; p = 0.01; OR: 2.0; p = 0.0289). A urethral or cervical infection (OR: 0.4; p = 0.0019) and not using a condom at one’s last sexual encounter (OR: 0.4; p = 0.0125) were found to be associated with ceftriaxone susceptibility for women.

### Determinants for azithromycin resistance (MIC > 0.5 mg/L)

#### Men who have sex with men

In the multivariable logistic analysis, 2012, 2013, and 2014 as year of diagnosis (aOR: 0.6; p = 0.0035; aOR: 0.6; p = 0.012; aOR: 0.7; p = 0.013, compared with 2011), being over 35 years of age (aOR: 0.7; p = 0.01) and a non-Dutch ethnicity (aOR: 0.1; p < 0.0001) were significantly associated with azithromycin susceptibility (Table 6).

**Table 6:** Univariable and multivariable analyses of azithromycin resistance^a^ of *Neisseria gonorrhoeae* in patients of Dutch sexually transmitted infection centres, by sex/sexual orientation, 2011–2015 (n = 6,893 isolates)

Characteristics	Univariable analyses OR (95%CI)			Multivariable analyses aOR (95%CI)		
	MSM	Heterosexual men	Women	MSM	Heterosexual men	Women
**Year of diagnosis**						
2012 (vs 2011)	0.6 (0.4–0.9)	2.3 (0.9–6.1)	0.9 (0.3–2.2)	0.6 (0.4–0.8)	2.0 (0.8–5.4)	0.8 (0.3–2.2)
2013 (vs 2011)	0.7 (0.5–1.0)	2.6 (1.0–7.1)	1.1 (0.5–2.6)	0.6 (0.4–0.9)	2.3 (0.8–6.3)	1.0 (0.4–2.3)
2014 (vs 2011)	0.9 (0.7–1.2)	2.4 (0.9–6.3)	1.0 (0.4–2.4)	0.7 (0.5–0.9)	1.8 (0.7–5.0)	0.8 (0.3–1.9)
2015 (vs 2011)	1.5 (1.1–1.9)	3.9 (1.5–10.0)	0.8 (0.3–2.0)	1.0 (0.7–1.3)	2.5 (0.9–6.6)	0.5 (0.2–1.3)
**Age (years)**						
25–35 (vs < 25)	0.7 (0.6–1.0)	2.4 (1.3–4.4)	1.5 (0.8–3.1)	0.9 (0.7–1.1)	2.5 (1.3–4.6)	NA^b^
≥ 35 (vs < 25)	0.6 (0.5–0.8)	2.7 (1.3–5.3)	2.7 (1.3–5.6)	0.7 (0.5–0.9)	2.6 (1.3–5.4)	NA^b^
**Other**						
Non-Dutch ethnicity^c^	0.1 (0.1–0.2)	0.3 (0.1–0.4)	0.4 (0.2–0.7)	0.1 (0.1–0.2)	0.3 (0.2–0.5)^d^	0.3 (0.2–0.6)^d^
CSW in last 6 months (MSM or women)	0.3 (0.1–0.9)	NA	1.9 (0.9–3.7)	NA^b^	NA^b^	NA^b^
Client of CSW in last 6 months	0.6 (0.2–2.0)	1.2 (0.6–2.6)	NA	NA^b^	NA^b^	NA^b^
Previous GO/chlamydia/syphilis	1.0 (0.8–1.3)	0.8 (0.4–1.8)	1.2 (0.6–2.4)	NA^b^	NA^b^	NA^b^
3–5 partners in last 6 months (vs 0–2)	0.8 (0.6–1.1)	1.8 (1.0–3.2)	0.6 (0.3–1.5)	NA^b^	2.2 (1.2–3.8)	NA^b^
≥ 6 partners in last 6 months (vs 0–2)	0.6 (0.5–0.8)	1.1 (0.5–2.3)	1.9 (1.0–3.6)	NA^b^	1.2 (0.5–2.5)	NA^b^
Casual partner	1.1 (0.9–1.3)	1.4 (0.8–2.4)	0.9 (0.5–1.6)	NA^b^	NA^b^	NA^b^
Notified of an STI by partner	0.9 (0.7–1.1)	0.6 (0.3–1.2)	0.9 (0.5–1.6)	NA^b^	NA^b^	NA^b^
No condom use at last contact	0.6 (0.5–0.8)	0.5 (0.3–0.9)	0.5 (0.3–1.0)	NA^b^	NA^b^	NA^b^
Urethral/cervical *Neisseria gonorrhoeae* infection	1.2 (1.0–1.4)	NA^e^	1.1 (0.5–2.5)	NA^b^	NA^b^	NA^b^
Anal *Neisseria gonorrhoeae* infection	1.1 (0.9–1.3)	NA^f^	0.7 (0.3–1.3)	NA^b^	NA^b^	NA^b^
Oral *Neisseria gonorrhoeae* infection	1.3 (1.0–1.6)	NA^f^	2.3 (1.3–4.1)	NA^b^	NA^b^	2.1 (1.2–3.9)^d^
Concurrent Ct infection	1.0 (0.8–1.3)	0.8 (0.5–1.4)	0.2 (0.1–0.5)	NA^b^	NA^b^	0.3 (0.1–0.5)

aOR: adjusted odds ratio; CI: confidence interval; CSW: commercial sex worker; EUCAST: European Committee on Antimicrobial Susceptibility Testing; GO: gonorrhoea; MIC: minimum inhibitory concentration; MSM: men who have sex with men; NA: not applicable; OR: odds ratio; STI: sexually transmitted infection.

^a ^Resistance according to EUCAST breakpoint (MIC > 0.5 mg/L).

^b^ Multivariable analyses using backward selection were only performed for variables with clinical and statistical importance (p < 0.2) in the univariable analyses.

^c ^A person with both parents born in the Netherlands was considered to be of Dutch ethnicity.

^d ^Factor not included in final model, excluding Amsterdam data.

^e ^Number of cases in the different categories not large enough to perform analysis.

^f^ No samples available for these sites in heterosexual men.

#### Heterosexual men

For heterosexual men, being older than 25 years (aOR: 2.5; p = 0.0049; aOR: 2.6; p = 0.0079) and having three to five partners (aOR: 2.2; p = 0.01, compared with 0–2 partners) were significantly associated with azithromycin resistance in the multivariable analysis. A non-Dutch ethnicity (aOR: 0.3, p < 0.0001) was significantly associated with azithromycin susceptibility.

#### Women

Having an oral gonorrhoea infection was significantly associated with azithromycin resistance in the multivariable analysis (aOR: 2.1; p = 0.0227). A non-Dutch ethnicity (aOR: 0.3, p = 0.0009) and a *C. trachomatis* co-infection (aOR: 0.3; p = 0.0004) were significantly associated with azithromycin susceptibility.

### Analyses without data from Amsterdam

Because there was a heavy influence of data submitted from Amsterdam (61.3% of all isolates between 2007 and 2015 came from Amsterdam) and it is known that a higher-risk population (73.6% MSM [26]) is being tested at the STI centre in Amsterdam, the analyses were repeated without these data and some differences were found. The multivariable analyses of MSM outside Amsterdam showed that being a client of a CSW was no longer associated with cefotaxime resistance (Table 4). For heterosexual men, receiving notification for an STI and not using a condom during the last sexual contact were no longer associated with susceptibility in the cefotaxime multivariable model. Urethral and anal infections were not associated with susceptibility to ceftriaxone in this multivariable model, and a non-Dutch ethnicity was no longer associated with decreased ceftriaxone susceptibility for MSM outside Amsterdam (Table 5). For azithromycin resistance, among women, there was no longer an association with an oral infection, and a non-Dutch ethnicity was no longer associated with azithromycin susceptibility (Table 6). A non-Dutch ethnicity was also not associated with susceptibility to azithromycin for heterosexual men.

## Discussion

Analysis of GRAS, the nationwide surveillance network focused on *N.* *gonorrhoeae* resistance, shows that resistance to ceftriaxone has not yet been reported in the Netherlands, despite the current increase of ceftriaxone resistance outside the Netherlands [25,29,30]. The proportion of isolates with a decreased susceptibility to ceftriaxone (MIC > 0.032 mg/L) varied over time; it increased from 3.5% in 2011 to 7.5% in 2014, but decreased again to 4.5% in 2015. No significant trend over time was found. 

Although azithromycin is not included in routine treatment of gonorrhoea in the Netherlands, there was a significant increase (p < 0.0001) in mean MIC for azithromycin, and resistance to azithromycin has increased to 10.9% in 2015. The proportion of isolates with high-level resistance to azithromycin (MIC  ≥ 2.0 mg/L) has remained stable over the years with 3.3% of isolates in 2011 and 3.0% of isolates in 2015, however, the increase in low-level azithromycin resistance (MIC = 1.0 mg/L) since 2014 could lead to an increase in higher resistant strains in the future. It could well be that a significant proportion of STI centre patients with gonorrhoea have undergone syndromic treatment for urethritis, including azithromycin, as the initial treatment procedure for urethritis with a low suspected risk of *N.* *gonorrhoeae* infection is 1,000 mg azithromycin [15]. This could explain low-level resistance, since it has recently been shown that *N. gonorrhoeae* strains cultured shortly after azithromycin treatment had increased MICs against azithromycin in comparison to other strains [31].

Cefotaxime was used as first-line treatment in the Netherlands up to 2006 and the proportion of isolates resistant to cefotaxime has decreased from 4.0% in 2007 to 1.9% in 2015. Interestingly, only 22.2% of strains with cefotaxime resistance also showed decreased susceptibility to ceftriaxone and alternatively, only 32.2% of strains with decreased susceptibility to ceftriaxone were resistant to cefotaxime, suggesting that different molecular mechanisms might be responsible for resistance against different 3G cephalosporins. On the other hand, there are no universally agreed breakpoints for decreased susceptibility to ceftriaxone and for cefotaxime resistance (e.g. the Clinical and Laboratory Standards Institute (CLSI) uses a higher breakpoint (MIC > 0.5 mg/L) [32] than EUCAST (MIC > 0.125 mg/L) for cefotaxime), so it cannot be ruled out that the breakpoints chosen in this study could also have contributed to discrepancies in the proportion of isolates resistant to cefotaxime that are also less susceptible to ceftriaxone and vice versa.

So far, these data support the Dutch decision to use ceftriaxone monotherapy for gonorrhoea instead of dual therapy with azithromycin; no resistance to ceftriaxone has been found since its introduction in 2006 while resistance to azithromycin is increasing.

When looking at determinants associated with decreased susceptibility of antibiotics used for treatment of gonorrhoea, differences were observed between people with different sexual behaviour. For women, the predominant determinant of decreased susceptibility of gonorrhoea was an oral infection; gonorrhoea in the pharynx was associated with resistance to azithromycin and cefotaxime. Also in MSM, oral infection was a risk factor for azithromycin resistance in the univariable analysis. Pharyngeal gonococcal infections are considered a potential reservoir for resistant isolates. It is thought that *N. gonorrhoeae* can become resistant through DNA acquisition from commensal *Neisseria* species, which could have been selected for by the lower efficacy of cephalosporins in the pharynx [24,33-35]. In the univariable analysis, isolates of women working as CSW were also associated with resistance to cefotaxime, as was the case in the univariable analyses on decreased susceptibility of ceftriaxone. However, working as a CSW was not significantly associated with gonococcal resistance in the multivariable analyses. Instead, having six or more sex partners was associated with resistance to cefotaxime, though number of sex partners can be considered a good proxy for sex work. A study on sexually transmitted infections among female sex workers in the Netherlands showed that of all gonorrhoea infections, oral infections were most commonly diagnosed (52.4%) [36]. Healthcare workers associated with the project also recognised that female CSW often do not use a condom when performing oral sex because they can charge a higher fee for this service. In this study, 56.2% of female CSW were diagnosed with oral infections vs 24.2% of women not working as sex workers. As pharyngeal gonorrhoea infections appear to be common among female CSW, emerging resistance of gonorrhoea in the pharynx in this risk group should continue to be carefully monitored.

Isolates of heterosexual men and women susceptible to cefotaxime, ceftriaxone or azithromycin were associated with a concurrent *C. trachomatis* infection. This has also been found by Cole et al. in isolates from Euro-GASP showing resistance to cefixime and ciprofloxacin [37]. Studies have been performed looking at the interaction between gonorrhoea and chlamydia [38-40] but a biological mechanism responsible for this manifestation has not been identified. A possible explanation for this association between resistance and a chlamydial co-infection could be a decrease in fitness of the *N. gonorrhoeae* when it has higher resistance levels and consequently preferential growth of chlamydia after co-infections with chlamydia and resistant *N.* *gonorrhoeae* strains. Bacteria are well known to be able to occupy a particular niche if they can better adapt to existing conditions. This mechanism is known e.g. in meticillin-resistant *Staphylococcus aureus* (MRSA) compared with non-resistant *Staphylococcus aureus* and various studies have demonstrated this relation between fitness and resistance levels [41-43].

Like Trecker et al. [44] and McLean et al. [45] but in contrast to Town et al. [46], our study showed a significant association with age: for heterosexual men, being older than 25 years of age was significantly associated with resistance to azithromycin and being older than 35 years of age was associated with decreased susceptibility to ceftriaxone. We also found a significant association with a higher number of sex partners (3–5 partners) and resistance to azithromycin among heterosexual men. The use of a condom during the last sexual contact by heterosexual men was associated with resistance to cefotaxime and in the univariable analysis with decreased susceptibility to ceftriaxone. A possible explanation could have to do with the time of observation: people visit the STI centre when there is an indication to do so. Possibly, heterosexual men who visit an STI centre suspect an STI and will have used condoms immediately before the visit. This reversed effect was previously shown in the Netherlands in MSM attaining a gonorrhoea infection [47]. 

Evidence of a decrease in azithromycin susceptibility in Europe has been reported in recent years [29,37,46], although contrary findings have also been reported. Wind et al. [26] report that azithromycin resistance remained stable around 1.2% in their Amsterdam study population, although the percentage of isolates with intermediate MICs (MIC > 0.25 to ≤ 0.5 mg/L) increased from 3.7% in 2012 to 15.6% in 2014. We only found an association between resistance to azithromycin and 2015 as year of diagnosis in univariable analyses, specifically among heterosexual men and MSM. We did, nevertheless, observe a significant increase in azithromycin MIC over time (p < 0.0001) and an increase in percentage of azithromycin resistance to 10.9% in 2015. The difference in azithromycin susceptibility between the national and Amsterdam data lies in the proportion of isolates with low-level resistance (MIC > 0.5 mg/L to ≤ 1 mg/L), which is higher in the Netherlands overall. This could possibly be explained by differences in laboratory techniques such as the use of different media for *N.* *gonorrhoeae* culture.

In addition, we found an association between a non-Dutch ethnicity and decreased ceftriaxone susceptibility in MSM, in contrast to the association of a non-Dutch ethnicity with azithromycin susceptibility among MSM. 

There are some limitations to this study. Firstly, for only 42.0% of all gonorrhoea diagnoses an isolate tested for susceptibility was available. This can be explained in part by negative cultures following an initial PCR diagnosis and either self-clearance or syndromic antibiotic treatment. In these patients, susceptibility testing is not possible. Overall, about half of the anal and cervical cultures was negative (50.3% and 51.1% respectively), the percentage of negative urethral cultures was slightly lower (39.8%) while the highest percentage of negative cultures was for oral cultures (72.2%). These negative cultures could also explain why patients with an isolate differed from patients without an isolate regarding their demographic characteristics. In addition, it was not possible to report the results of more than one culture per patient to GRAS.

Secondly, isolates were selected based on new consultations. Since June 2014, an ID number has been available in SOAP and analysing the data from June 2014 until December 2015, we found that some patients were included multiple times (15.2%). This could have influenced our results if a strain was included more than once due to patients being re-infected by the same untreated partner with the same strain.

Thirdly, there is a heavy influence of data submitted from Amsterdam; 61.3% of all isolates between 2007 and 2015 came from Amsterdam. Analyses without the data from Amsterdam gave partially different results; this is probably due to the higher-risk population (73.6% MSM) that is being tested at the STI centre in Amsterdam [26].

Furthermore, no form of molecular typing was performed in this study. The results on possible gonorrhoea resistance were only confirmed with an Etest. Possible links to known clusters or clones could therefore not be identified. Finally, we do not know the situation regarding resistance of *N. gonorrhoeae* at the general practitioner (GP). Because of the logistics associated with GRAS, GPs do not participate in this surveillance, and GRAS surveillance assumes that resistance is most likely to emerge and spread first in the high-risk population seen at the STI centres.

In conclusion, resistance to ceftriaxone has not been detected yet in the Netherlands and no significant trend in MICs over time has been found; however, a significant increase in MIC for azithromycin over time has been identified. The current understanding of possible determinants for antimicrobial resistance in *N. gonorrhoeae* in the Netherlands is limited and identifying risk factors for AMR infections could help determine evidence-based risk groups and subsequent focused treatments or public health intervention strategies. Targeted surveillance of new resistance patterns and insights into sexual networks and determinants is essential. To continue improving the AMR surveillance for gonorrhoea in the Netherlands, GRAS has been expanded allowing for more than one culture per patient to be reported starting in 2016. Further optimisation of GRAS could be the inclusion of culture results from GPs. In addition, the development of new treatment strategies and reassessment of older antimicrobial agents is necessary to prevent severe public health consequences.
